# Renal protective effect of ellipticine against streptozotocin induced diabetic nephropathy in rats via suppression of oxidative stress and inflammatory mediator

**DOI:** 10.1590/acb385623

**Published:** 2023-12-04

**Authors:** Jun Li, Yu Xie, Jimei Sun, Fan Bai, Shaik Althaf Hussain, Venkata Subba Reddy Gangireddygari, Xiaolan Jiang

**Affiliations:** 1 The First People’s Hospital of Yunnan Province – Department of Nephrology – The Affiliated Hospital of Kunming University of Science and Technology – Kunming – China.; 2 The First People’s Hospital of Kunming – Department of Nephrology – Kunming – China.; 3 King Saud University – College of Science – Department of Zoology – Riyadh – Saudi Arabia.; 4 National Institute of Horticultural and Herbal Science – Plant Virus Research – Horticultural and Herbal Crop Environment Division – Rural Development Administration – Wanju – Republic of Korea.

**Keywords:** Cytokines, Diabetes Mellitus, Inflammation, Fibrosis, Antioxidants

## Abstract

**Purpose:**

Diabetes mellitus is a serious health problem worldwide, and diabetic nephropathy is the complication. The diabetic nephropathy considerably enhances the oxidative stress, glycation, lipid parameters and inflammatory reaction. Ellipticine has potent free radical scavenging and anti-inflammatory effect.

**Methods:**

In the current study, our objectives were to thoroughly examine the renal protective effects of ellipticine in a rat model of streptozotocin (STZ)-induced diabetic nephropathy (DN) and to elucidate the underlying mechanisms involved. For the induction of diabetic nephropathy, streptozotocin (50 mg/kg) was used, and rats were separated into groups and given varying doses of ellipticine (2.5, 5 and 7.5 mg/kg). The body weight, and renal weight were estimated. The inflammatory cytokines, renal biomarkers, inflammatory antioxidant, and urine parameters were estimated.

**Results:**

Result showed that ellipticine considerably enhanced the body weight and reduced the renal tissue weight. Ellipticine treatment significantly (P < 0.001) repressed the level of blood urea nitrogen, serum creatinine, uric acid, blood glucose and altered the lipid parameters. Ellipticine significantly (P < 0.001) repressed the level of malonaldehyde and boosted the glutathione, catalase, superoxide dismutase, and glutathione peroxidase. Ellipticine treatment significantly (P < 0.001) reduced the inflammatory cytokines and inflammatory mediators.

**Conclusions:**

Ellipticine could be a renal protective drug via attenuating the inflammatory reaction, fibrosis and oxidative stress in streptozotocin induced rats.

## Introduction

Diabetes mellitus (DM) is a progressively metabolic syndrome in which metabolism of fat, protein and glucose induces the serious injury in the renal tissue^1^. The incidence of DM in Asia increases day by day. In Asian continent country, especially China, 11.6% people suffered from the DM, and around one third patient developed the diabetic nephropathy (DN)^2^. The major microvascular dysfunction of DM such as nephropathy, retinopathy and neuropathy are commonly observed in the diabetic patients^3^^,^ 4.

DN is a significant consequence of DM that leads to renal failure^5^. In the last two decades, the incidence of DN suggestively raised^6^. Yin et al. showed that more than 100 million people suffer from the DN, and this number is going to double in the current decades^7^. The major cause of DN is hyperglycemia, which causes renal damage and glomerular dysfunction by suppressing insulin secretion (type I DM) or decreasing tissue sensitivity to insulin (type II DM)^8^^,^ 9. Because the progression of end-stage renal disease is irreversible, it is essential to find ways to slow the progression of kidney damage^5^^,^ 10. The oxidative stress, genetic, hemodynamic, inflammatory and metabolic factors involved in the pathogenesis of DN^11^^,^ 12.

The activation of transforming growth factor-β (TGF-β) and increased oxidative stress are well recognized to play a role in the expansion of DN^13^^,^ 14. The balance between the endogenous antioxidant defense system enzymes such as malonaldehyde (MDA), nicotinamide adenine dinucleotide phosphate (NADPH), superoxide dismutase (SOD) and oxidative stress is commonly used for the determination of degree of oxidative stress^15^–^17^. The increased level of reactive oxygen species (ROS) boosted the TGF-β expression via activation of signal transduction cascade involving nuclear receptor peroxisome proliferator activated receptor-γ (PPAR-γ) and mitogen activated protein kinase (MAPK)^14^^,^ 18.

The medicinal plant has long history to treat the diseases from the ancient times. The major phytoconstituents such as flavonoids, polysaccharides, alkaloids, tannins and steroids are rich medicinal plants healing various diseases^19^^,^ 20. In the last few decades, the demands of the herbal plants increase due to more potential effect with less side/toxic effects. Ellipticine (alkaloid) isolated from the *Ochrosia elliptica* and belongs with the Apocynaceae family^21^. Ellipticine demonstrates anticancer effect, and already several derivatives of ellipticine are under the clinical trials^22^–^24^. Ellipticine demonstrated anti-inflammatory effect against the lipopolysaccharide induces macrophages via targeting the JNK/AP-1 signaling pathway^24^. Current research has shown the synergistic effect of ellipticine in epithelial cells and that it alleviates the acute pancreatitis related with the acute lung injury in rats^21^^,^ 23 ^,^ 24. The anticancer activity of ellipticine was demonstrated through antioxidant actions^25^^,^ 26.

Considering the antioxidant and anti-inflammatory effects of ellipticine, this study aimed to assess the renal protective potential of ellipticine in a STZ induced DN model.

## Methods

### Experimental animals

Free-pathogen Sprague Dawley rats (aged 8–10 weeks, 200 ± 25 g, male) were procured from the experimental animal house and kept in the standard laboratory condition, such as 20 ± 5°C, 60–75% of relative humidity and 12/12 dark/light cycle. All the experimental investigation was performed according to the institutional animal care and use committee of the university.

### Toxicant and test drug preparation

DM was induced with an intraperitoneal injection of STZ (50 mg/kg)^27^. Briefly, the toxicant was diluted in the 10 mM citrate buffer, and pH = 4.5 was maintained. Test drug prepared the different doses (2.5, 5 and 7.5 mg/kg)^28^ via suspended into the carboxyl methylcellulose.

### Experimental procedure

After injecting the toxicant (STZ) for inducing the DM, the blood glucose level was estimated after seven days, and rats with more than 250 mg/dL were considered as diabetic^15^^,^ 17. The rats were divided into the following groups:

- Group I: normal;
- Group II: STZ;
- Group III–V: STZ + ellipticine (2.5, 5 and 7.5 mg/kg).

Each group contained six rats and got the mentioned medication once a day for 56 days by oral delivery. Rats’ body weight, food, and water intake were measured at regular intervals throughout the trial. At the end of the study, the urine sample of all group rats were collected using the metabolic cage. Before the sacrifice, blood samples from all groups of rats were taken from the abdominal aorta’s arterial blood. The rats were sacrificed by cervical dislocation, and a kidney sample was taken and frozen in liquid nitrogen right away.

### Biochemical parameters

The standard kits were used for the estimation the uric acid, bilirubin, creatinine, urea, and albumin via following the manufacture’s instruction (Nanjin Jiancheng Bioengineering Institute, Nanjing, China).

Advanced glycation end products (AGEs) and 8-Hydroxydeoxyguanosine (8-OHdG) level were estimated via using the enzyme-linked immunosorbent assay (ELISA) kits.

The lipid parameters such as triglyceride (TG), high-density lipoprotein (HDL) were estimated using the kits.The previous reported method was used for the estimation of low-density lipoprotein (LDL) and very high-density lipoprotein.

The oxidative stress parameters viz., catalase (CAT), , glutathione (GSH), superoxide dismutase (SOD), glutathione peroxidase (GPx), and MDA were examined using the manufacture’s instruction

The inflammatory cytokines viz., tumor necrosis factor-? (TNF-?), interleukin-6 (IL-6) and interleukin-1? (IL-1?) were examined using the manufacture’s instruction

Inflammatory parameters such as cyclooxygenase-2 (COX-2), nuclear kappa B factor (NF-κB), prostaglandin (PGE_2_), and TGF-β1 were estimated using the manufacture’s instruction (Nanjin Jiancheng Bioengineering Institute, Nanjing, China).

### Statistical analysis

All the data of this study was presented as mean ± standard error of the mean (SEM), and GraphPad Prism 8 software (San Diego, CA, United States of America) was used for the statistical analysis. One-way analysis of variance (ANOVA) followed by Dunnett’s test was used for the statistical comparisons between the groups, and P < 0.05 was consider as the statistically significant.

## Results

### Body weight, renal weight, and renal index

**Figure 1:**
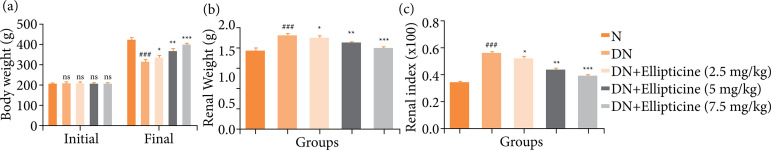
Effect of ellipticine on the body weight, renal weight, and renal index in streptozotocin induced DN rats. **(a)** Body weight, **(b)** renal weight, and **(c)** renal index. Data are presented as mean ± standard error from six rats in each group. DN group rats were compared with normal rats. Ellipticine treated group rats were compared with DN group rats.

NC: normal control; DN: diabetic nephropathy; ^###^P < 0.001 = extreme significant; *P < 0.05 = significant, **P < 0.01 = more significant; ***P < 0.001 = extreme significant. Source: elaborated by the authors.

When compared to normal and testing group rats, STZ induced DN rats demonstrated a decrease in body weight. DN group rats treated with the ellipticine significantly (P < 0.001) increased the body weight. Ellipticine (7.5 mg/kg) treated rats improved the weight and similar to the normal rats (Fig. 1a).

STZ-induced DN resulted in increase in renal weight (Fig. 1b), as well as a higher renal index (Fig. 1c). Ellipticine treatment considerably (P < 0.001) reduced the renal weight and renal index.

### Glucose and insulin level

**Figure 2:**
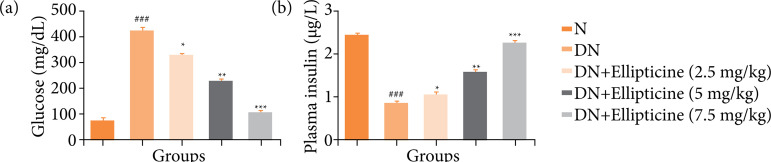
Effect of ellipticine on the blood glucose and insulin level index in streptozotocin induced DN rats. **(a)** Blood glucose, and **(b)** plasma insulin. Data are presented as mean ± standard error from six rats in each group. DN group rats were compared with normal rats. Ellipticine treated group rats were compared with DN group rats.

NC: normal control; DN: diabetic nephropathy; ^###^P < 0.001 = extreme significant; *P < 0.05 = significant, **P < 0.01 = more significant; ***P < 0.001 = extreme significant. Source: elaborated by the authors.

STZ induced DN rats demonstrated the enhancement of glucose level (Fig. 2a) and suppression of plasma insulin (Fig. 2b). STZ induced DN rats treated with the ellipticine considerably (P < 0.001) downregulated the glucose level and boosted the insulin level.

### Advanced glycation end products and 8-hydroxydeoxyguanosine

**Figure 3:**
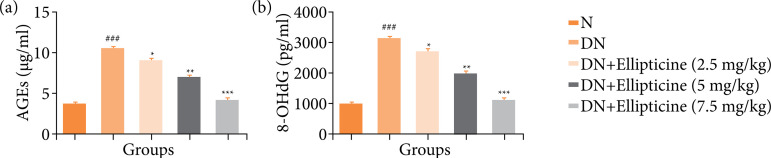
Effect of ellipticine on the AGEs and 8-OHdG in streptozotocin induced DN rats. **(a)** AGEs, and **(b)** 8-OHdG. Data are presented as mean ± standard error from six rats in each group. DN group rats were compared with normal rats. Ellipticine treated group rats were compared with DN group rats.

NC: normal control; DN: diabetic nephropathy; ^###^P < 0.001 = extreme significant; *P < 0.05 = significant, **P < 0.01 = more significant; ***P < 0.001 = extreme significant; AGEs: advanced glycation end products; 8-OHdG: 8-hydroxydeoxyguanosine. Source: elaborated by the authors.

During the condition, the level of AGEs and 8-OHdG boosted, and similar result was observed in the DN group rats. Ellipticine treatment significantly (P < 0.001) reduced the AGEs (Fig. 3a) and 8-OHdG (Fig. 3d).

### Renal parameters

**Figure 4:**
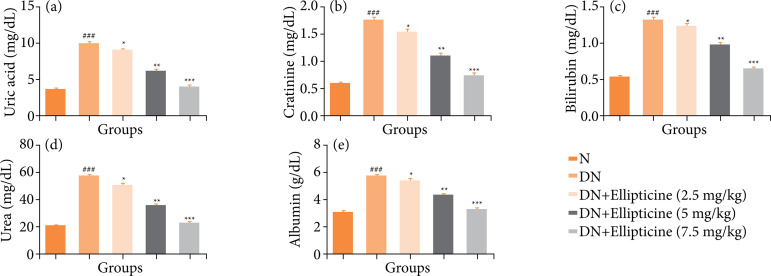
Effect of ellipticine on the renal parameters in streptozotocin induced DN rats. **(a)** uric acid, **(b)** creatinine, **(c)** bilirubin, **(d)** urea, and **(e)** albumin. Data are presented as mean ± standard error from six rats in each group. DN group rats were compared with normal rats. Ellipticine treated group rats were compared with DN group rats.

NC: normal control; DN: diabetic nephropathy; ^###^P < 0.001 = extreme significant; *P < 0.05 = significant, **P < 0.01 = more significant; ***P < 0.001 = extreme significant. Source: elaborated by the authors.

DN group rats demonstrated the increased level of uric acid (Fig. 4a), creatinine (Fig. 4b), bilirubin (Fig. 4c), urea (Fig. 4d), and albumin (Fig. 4e), and ellipticine treatment remarkably (P < 0.001) repressed the level of renal parameters.

### Lipid parameters

**Figure 5:**
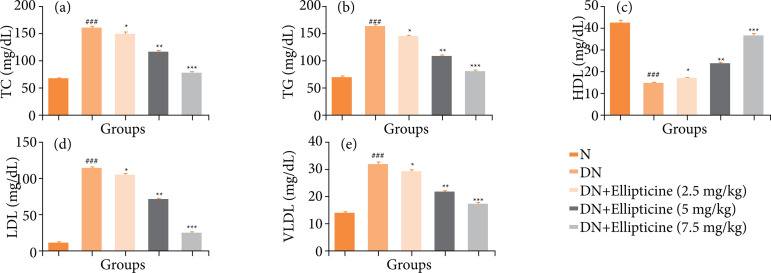
Effect of ellipticine on the lipid parameters in streptozotocin induced DN rats. **(a)** TC, **(b)** TG, **(c)** HDL, **(d)** LDL, and **(e)** VLDL. Data are presented as mean ± standard error from six rats in each group. DN group rats were compared with normal rats. Ellipticine treated group rats were compared with DN group rats.

NC: normal control; DN: diabetic nephropathy; ^###^P < 0.001 = extreme significant; *P < 0.05 = significant, **P < 0.01 = more significant; ***P < 0.001 = extreme significant; TC: total cholesterol; TG: triglycerides; HDL: high-density lipoprotein; VLDL: very low-density lipoprotein. Source: elaborated by the authors.

Figure 5 demonstrated the level of lipid parameters. DN group rats exhibited the enhanced level of TC, TG, LDL, very low-density lipoprotein (VLDL) and repressed level of HDL. Ellipticine remarkably (P < 0.001) altered the level of lipid parameters.

### Antioxidant parameters

**Figure 6:**
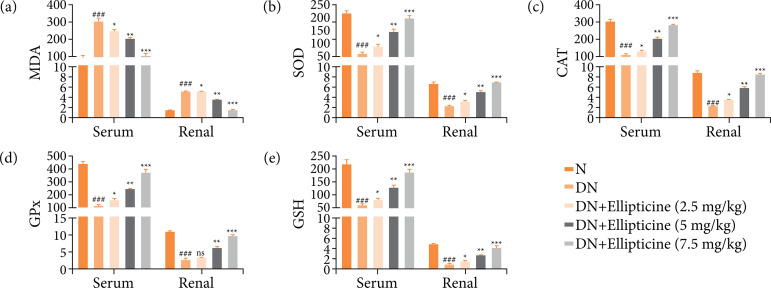
Effect of ellipticine on the antioxidant parameters in streptozotocin induced DN rats. **(a)** MDA, **(b)** SOD, **(c)** CAT, **(d)** GPx and **(e)** GSH. Data are presented as mean ± standard error from six rats in each group. DN group rats were compared with normal rats. Ellipticine treated group rats were compared with DN group rats.

NC: normal control; DN: diabetic nephropathy; ^###^P < 0.001 = extreme significant; *P < 0.05 = significant, **P < 0.01 = more significant; ***P < 0.001 = extreme significant; MDA: malonaldehyde; SOD: superoxide dismutase; CAT: catalase; GPx: glutathione peroxidase; GSH: glutathione. Source: elaborated by the authors.

DN group rats exhibited the augmented level of MDA (Fig. 6a) and repressed the level of SOD (Fig. 6b), CAT (Fig. 6c), GPx (Fig. 6d), and GSH (Fig. 6e) in the serum and renal tissue. Ellipticine (P < 0.001) altered the level of antioxidant parameters.

### Inflammatory cytokines

**Figure 7:**
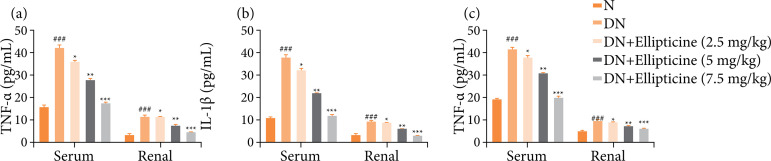
Effect of ellipticine on the inflammatory cytokines in streptozotocin induced DN rats. **(a)** TNF-α, **(b)** IL-1β, and **(c)** IL-6. Data are presented as mean ± standard error from six rats in each group. DN group rats were compared with normal rats. Ellipticine treated group rats were compared with DN group rats.

NC: normal control; DN: diabetic nephropathy; ^###^P < 0.001 = extreme significant; *P < 0.05 = significant, **P < 0.01 = more significant; ***P < 0.001 = extreme significant; TNF-α: tumour necrosis factor-α; IL: interleukin. Source: elaborated by the authors.

DN group rats exhibited the improved level of inflammatory cytokines TNF-α (Fig. 7a), IL-6 (Fig. 7b), and IL-1β (Fig. 7c), and ellipticine significantly (P < 0.001) reduced the level of inflammatory cytokines.

### Inflammatory mediators

**Figure 8:**
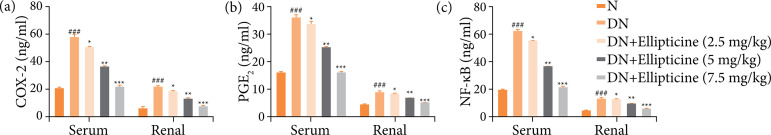
Effect of ellipticine on the inflammatory parameters in streptozotocin induced DN rats. **(a)** COX-2, **(b)** PGE_2_, and **(c)** NF-κB. Data are presented as mean ± standard error from six rats in each group. DN group rats were compared with normal rats. Ellipticine treated group rats were compared with DN group rats.

NC: normal control; DN: diabetic nephropathy; ^###^P < 0.001 = extreme significant; *P < 0.05 = significant, **P < 0.01 = more significant; ***P < 0.001 = extreme significant; COX-2: cyclooxygenase-2; PGE_2_: prostaglandin; NF-κB: nuclear kappa B factor. Source: elaborated by the authors.

DN group rats demonstrated the enhanced inflammatory mediators includes COX-2 (Fig. 8a), PGE_2_ (Fig. 8b), and NF-κB (Fig. 8c), and ellipticine significantly (P < 0.001) suppressed the inflammatory mediators. Ellipticine (7.5 mg/kg) exhibited the maximum reduction.

**Figure 9:**
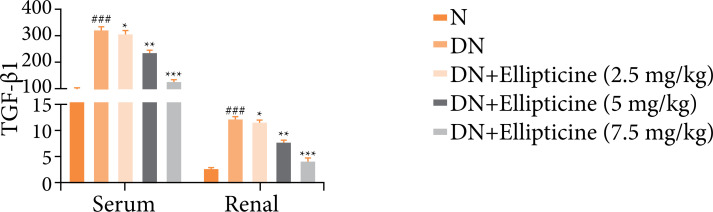
Effect of ellipticine on the TGF-β1 in streptozotocin induced DN rats. Data are presented as mean ± standard error from six rats in each group. DN group rats were compared with normal rats. Ellipticine treated group rats were compared with DN group rats.

NC: normal control; DN: diabetic nephropathy; ^###^P < 0.001 = extreme significant; *P < 0.05 = significant, **P < 0.01 = more significant; ***P < 0.001 = extreme significant; TGF-β: transforming growth factor-β. Source: elaborated by the authors.

DN group rats exhibited level of TGF-β1 in the serum and renal tissue. Ellipticine treated rats significantly (P < 0.001) suppressed the TGF-β1 level in the serum and renal tissue (Fig. 9).

## Discussion

DN with the glomerulosclerosis and proteinuria is a complication of DN that develops in 31% patients of diabetes (type I)^3^^,^ 4. It is well known that increase blood glucose level boosts the production of ROS and also suppresses the proximal tubular function and causes the podocytes apoptosis^29^.

We administered the ellipticine to STZ induced DN rats and estimated the renalprotective and antidiabetic effect. End of the experimental study, ellipticine considerably suppressed the glucose level, cytokines, AGEs, inflammatory mediators, renal biomarkers, and oxidative stress parameters. Ellipticine considerably repressed the cytokines (estimated in term of TNF-?, IL-1?, Il-6), inflammatory mediators (COX-2, PGE_2_, NF-κB), antioxidant parameters (improved the level of SOD, CAT, GSH, GPx and suppressed the level of MDA). Previous literature suggests that oxidative stress is the common phenomenon in DN induced via high blood glucose level^30^^,^ 31. Hyperglycemia boosts the ROS production and induces the oxidative stress, which result in loss of renal function.

STZ is a commonly used toxicant for induction the diabetes (type I and type II) in the rodents^16^^,^ 32. It is a widely accepted model due to necrotic effect on the pancreatic β-cells and reduction of insulin production/secretion from the cells^15^. The DN rats in this study had an elevated glucose level, which was lowered by ellipticine. Hyperglycemia is well recognized to increase the risk of renal injury, and our medicine lowers glucose levels, suggesting that ellipticine may also protect renal tissue from the damage caused by high glucose. The body weight of STZ-induced DN rats was suppressed due to persistent hyperglycemia^33^. Ellipticine treatment improved the body weight, indicating the protection against the muscle injury induced by the hyperglycemia. DN rats also exhibited the increased renal tissue weight as compared to the normal and treated group rats showing to hypertrophy. Ellipticine treatment considerably suppressed the renal tissue weight and brought it back to normal level, suggesting the protection against the renal hypertrophy.

DN is induced by the multiple mechanisms, and the researcher chooses the drugs that have the multiple pharmacological action^34^^,^ 35. DM led to hypercholesterolemia, hypertriglyceridemia, and fatty liver. Kumar et al. showed that the high cholesterol level is associated with the high glucose level^15^^,^ 17. In this experimental study, DN group rats exhibited the boosted level of TC, TG, LDL, and VLDL, and ellipticine treatment considerably restored the level of lipid parameters.

Hypoalbuminemia is considered as the gold marker for the strongly predictor of patient death due to renal failure^36^. Albumin is the significant protein marker in nephrotic urine, and during the DN, the level of albumin reduced in the serum and boosted the level of albumin in the urine showed the albuminuria, which is related to dysfunction of kidney function^37^. However, ellipticine treatment considerably normalized the level of albumin and suggested the preventive effect against microalbuminuria. Other markers of the DN are serum creatinine (SCr) and blood urea nitrogen (BUN). During the DN disease, the level of SCr decreased in the urine and suggested the expansion of DN.

According to the findings, ROS plays an essential role in the pathogenesis of DN^38^^,^ 39. Due to the high blood glucose level, it enhances the production of free radical, which further induces the oxidative stress and start the accumulation of ROS via incomplete oxidation of glucose^5^^,^ 38. Because renal tissue is more vulnerable to increase glucose in the circulation, ROS play a remarkable role in the progression of diseases such as diabetic nephropathy^38^^,^ 39. Previous research suggested that the oxidative stress suppress the renal tissue function, and the researcher used the antioxidant drug to improve the level of renal tissue^37^. The antioxidant drugs contributed to enhanced the renal function and also provided the protection to renal tissue against the oxidative injury^40^.

It is widely recognized that elevated glucose levels play a significant role in mediating oxidative stress, which in turn contributes to the progression and pathogenesis of DN^37^. During oxidative stress, the production of lipid peroxidation products, including hydroxyl radicals, ketone groups, and MDA, becomes initiated. These compounds play a significant role in the progression of renal disease by affecting the oxidation of proteins and amino acids^5^^,^ 30. During the DN, the activity of endogenous antioxidant enzymes is suppressed and the load of ROS in the tissue increases.

SOD, as the primary endogenous antioxidant, plays a crucial role in clearing free radicals and is also involved in protecting cells from injury^41^. CAT is the tetrameric hemin enzyme which breakdowns the hydrogen peroxide (H_2_O_2_) into the superoxide radical (O_2_) and H_2_O^42^. GPx, a selenium-containing enzyme, functions to break down lipid peroxides and H_2_O_2_ by utilizing GSH. This enzymatic action serves to protect cells from the damaging effects of free radicals^43^. Indeed, GSH is an additional free radical scavenger and serves as a co-substrate for the activity of GPx. Moreover, GSH plays a crucial role in various enzymatic reactions within the cell, contributing to its antioxidant and detoxification functions^44^. GST (glutathione dependent cytosolic enzyme), which protects the cells from the ROS injury, induces in the cells^33^. DN group rats exhibited the induction of oxidative stress (boosted the level of MDA and suppressed level of SOD, CAT, GSH, GPx) in the renal tissue, and ellipticine treatment considerably suppressed the oxidative stress via improving the level of endogenous antioxidant.

During the DN disease, the hemokinesis imbalance increases and oxidative stress induces the infiltration of macrophages, T-lymphocytes and white blood cells, which contributed to the production/secretion of inflammatory cells like INF-γ, Il-1, IL-1β and TNF-α^7^. These inflammatory cytokines could start the production of chemotactic factor in the renal tissue and also induce the infiltration of inflammatory cells^45^^,^ 46. This process may boost the serve injury in the renal tissue. During the renal damage, TNF-α boost the various chemotactic and inflammatory factors, which lead the expansion of disease and inflammatory reaction in the tissue^46^^,^ 47. IL-6 may also induce the expression of factors related with the fibrosis, which resultant causes the fibrosis and hypertrophy in the renal tissue^48^.

It also alters the permeability of renal endothelium, which in turn contributes to the expression of fibronectin and enhances the thickness of the glomerular basement membrane^7^. In this experimental study, we observed an increased level of inflammatory cytokines in renal tissues, and the treatment with ellipticine significantly suppressed these inflammatory cytokine levels. Based on these results, we can conclude that ellipticine suppresses the infiltration of inflammatory cells and thereby triggers an improvement in renal function. The formation of AGEs plays a pivotal role in the progression of DN. The irreversible formation of AGEs affects the lipids and proteins that induce the injury in the renal tissue and blood vessels. Studies showed that the AGEs are commonly found in all tissue of the body, and the renal tissue are more susceptible for the formation of AGEs and other tissues. In this experimental study, the level of AGEs considerably boosted observed in the DN group rats. Treatment with ellipticine significantly reduced the levels of AGEs, suggesting its potential renal protective and tissue-protecting effects.

It is well known that chronic inflammatory reaction boosted during the DN, and it plays a crucial role in the pathogenesis of DN^6^^,^ 49. Chronic inflammation expansion the renal disease along with addition of inflammatory molecules includes proinflammatory cytokines and adhesion molecules. NF-κB is the central signaling pathway in inflammation, and it is usually activated during the inflammatory reaction^50^–^52^. The activation of NF-κB can cause the transcription of various cytokines. Meanwhile, stimulation of monocyte chemoattractant protein-1 (MCP-1) can speed up macrophage migration into the kidneys, and its activation aggravates the accumulation of extracellular matrix (ECM) via the production of TGF-β^53^^,^ 54. STZ induced DN exhibited the boosted level of NF-κB, MCP-1 and TGF-β, and ellipticine treatment considerably suppressed the level of inflammatory mediators, suggesting the anti-inflammatory effect.

## Conclusion

The current study demonstrated that oral administration of ellipticine resulted in several positive effects, including a decrease in glucose levels, a reduction in renal weight, an increase in body weight, and normalization of the lipid profile. Moreover, ellipticine considerably suppressed renal biomarkers, inflammatory cytokines, and inflammatory parameters. Additionally, it induced alterations in the levels of antioxidant parameters. Based on our result, we can conclude ellipticine exhibited antihyperglycemic, anti-inflammatory, antiglycation and antioxidant effects. Furthermore, more work is necessary to scrutinize the underlying mechanism at molecular and cellular level.

## Data Availability

All the data available on the request from the corresponding author.
